# Supplementary material for: Economic evaluation of dialysis treatment in end-stage renal disease patients with fluid and sodium overload: Evidence from a randomized controlled trial in Thailand
Source: PLoS One. 2025 Nov 5;20(11):e0335749. doi: 10.1371/journal.pone.0335749 (PMC12588515; doi:10.1371/journal.pone.0335749)
Supplement: S1 Table — (DOCX) [file pone.0335749.s001.docx]

**Table S1 Baseline Characteristics of Study Participants.**

| **Variable** | **CAPD (n=60)** | **CAPD+ICO (n=60)** | **APD (n=60)** |
| --- | --- | --- | --- |
| Age (years) | 54.4 | 54.3 | 54.3 |
| Body Surface Area (m²) | 1.69 | 1.71 | 1.69 |
| Body Mass Index (BMI) | 25.3 | 25.2 | 26.3 |
| Systolic Blood Pressure  (mmHg) | 165 | 161 | 157 |
| Diastolic Blood Pressure (mmHg) | 89 | 88 | 86 |
| ACEI or ARB use (%) | 80 | 83 | 78 |
| RRT duration (months) | 41 | 35 | 38 |
| Urine volume >100 ml (%) | 22% | 23% | 32% |
| CAPD volume/day (ml) | 8,000 | 8,000 | 8,000 |
| Charlson Comorbidity Index (median) | 5 | 5 | 5 |
| Diabetes Mellitus (%) | 43% | 52% | 53% |
| MACE plus (%) | 42% | 28% | 33% |
| Peritonitis rate (episodes/year) | 0.20 | 0.23 | 0.18 |
| Hemoglobin (g/dL) | 9.5 | 9.9 | 9.8 |
| HbA1c | 5.8 | 6.0 | 6.4 |
| Serum albumin (g/dL) | 3.3 | 3.5 | 3.4 |
| LDL cholesterol (mg/dL) | 107 | 115 | 112 |
| Triglyceride (mg/dL) | 113 | 129 | 126 |
| Calcium (mg/dL) | 9.0 | 9.0 | 9.0 |
| Phosphorus (mg/dL) | 4.9 | 4.9 | 4.5 |
| iPTH (pg/mL) | 337 | 280 | 302 |
| CRP (mg/dL) | 3.0 | 1.7 | 2.7 |
| CA125 (U/mL) | 21.3 | 26.7 | 22.3 |
| Weekly Total Kt/Vurea | 1.69 | 1.67 | 1.77 |
| Weekly Total CCr (L) | 48.7 | 44.8 | 48.2 |
| Dcr4hr/Pcr2hr | 0.73 | 0.70 | 0.71 |
| Sodium dip (mmol/L) | 6.8 | 6.0 | 7.0 |

ACEI indicates angiotensin converting enzyme inhibitor; ARB, angiotensin receptor blocker; RRT, renal replacement therapy; MACE plus, major adverse cardiovascular events plus peripheral vascular disease; iPTH, intact parathyroid hormone; Dcr4hr/Pcr2hr, Dialysate creatinine at 4 hours/Plasma creatinine at 2 hours
