# Supplementary material for: Economic evaluation of dialysis treatment in end-stage renal disease patients with fluid and sodium overload: Evidence from a randomized controlled trial in Thailand
Source: PLoS One. 2025 Nov 5;20(11):e0335749. doi: 10.1371/journal.pone.0335749 (PMC12588515; doi:10.1371/journal.pone.0335749)
Supplement: S2 Table — (DOCX) [file pone.0335749.s002.docx]

**Table S2** **Model Parameters.**

| **Parameters** | **Distribution** | **Mean** | **SE** | **References** |
| --- | --- | --- | --- | --- |
| **Transitional probability** |  |  |  |  |
| Probability of transition from CAPD to APD in year 1 | Beta | 0.0200 | 0.0020 | [1] |
| Probability of transition from CAPD to APD in year 2 | Beta | 0.0500 | 0.0050 | [1] |
| Probability of transition from CAPD to APD in year 3 onward | Beta | 0.0500 | 0.0050 | [1] |
| Probability of transition from CAPD to death in year 1 | Beta | 0.1667 | 0.0167 | [1] |
| Probability of transition from CAPD to death in year 2 | Beta | 0.2449 | 0.0245 | [1] |
| Probability of transition from CAPD to death in year 3 onward | Beta | 0.2424 | 0.0242 | [1] |
| Probability of transition from CAPD to HD in year 1 | Beta | 0.0167 | 0.0017 | [1] |
| Probability of transition from CAPD to HD in year 2 | Beta | 0.0408 | 0.0041 | [1] |
| Probability of transition from CAPD to HD in year 3 onward | Beta | 0.1515 | 0.0152 | [1] |
| Probability of transition from CAPD to CAPD+ICO in year 1 | Beta | 0.0200 | 0.0020 | [1] |
| Probability of transition from CAPD to CAPD+ICO in year 2 | Beta | 0.0500 | 0.0050 | [1] |
| Probability of transition from CAPD to CAPD+ICO in year 3 onward | Beta | 0.0500 | 0.0050 | [1] |
| Probability of transition from CAPD to KT in year 1 | Beta | 0.0167 | 0.0017 | [1] |
| Probability of transition from CAPD to KT in year 2 | Beat | 0.0167 | 0.0017 | [1] |
| Probability of transition from CAPD to KT in year 3 onward | Beta | 0.0167 | 0.0017 | [1] |
| Probability of transition from APD to CAPD in year 1 | Beta | 0.0500 | 0.0050 | [1] |
| Probability of transition from APD to CAPD in year 2 | Beta | 0.0500 | 0.0050 | [1] |
| Probability of transition from APD to CAPD in year 3 onward | Beta | 0.0500 | 0.0050 | [1] |
| Probability of transition from APD to Death in year 1 | Beta | 0.2167 | 0.0217 | [1] |
| Probability of transition from APD to Death in year 2 | Beta | 0.3171 | 0.0317 | [1] |
| Probability of transition from APD to Death in year 3 onward | Beta | 0.2500 | 0.0250 | [1] |
| Probability of transition from APD to HD in year 1 | Beta | 0.1000 | 0.0100 | [1] |
| Probability of transition from APD to HD in year 2 | Beta | 0.0732 | 0.0073 | [1] |
| Probability of transition from APD to HD in year 3 onward | Beta | 0.2917 | 0.0292 | [1] |
| Probability of transition from APD to CAPD+ICO in year 1 | Beta | 0.0500 | 0.0050 | [1] |
| Probability of transition from APD to CAPD+ICO in year 2 | Beta | 0.0500 | 0.0050 | [1] |
| Probability of transition from APD to CAPD+ICO in year 3 onward | Beta | 0.0500 | 0.0050 | [1] |
| Probability of transition from APD to KT in year 1 | Beta | 0.0167 | 0.0017 | [1] |
| Probability of transition from APD to KT in year 2 | Beta | 0.0167 | 0.0017 | [1] |
| Probability of transition from APD to KT in year 3 onward | Beta | 0.0167 | 0.0017 | [1] |
| Probability of transition from CAPD+ICO to APD in year 1 | Beta | 0.00250 | 0.0003 | [1] |
| Probability of transition from CAPD+ICO to APD in year 2 | Beta | 0.00200 | 0.00020 | [1] |
| Probability of transition from CAPD+ICO to APD in year 3 onward | Beta | 0.00200 | 0.00020 | [1] |
| Probability of transition from CAPD+ICO to CAPD in year 1 | Beta | 0.0008 | 0.0001 | [1] |
| Probability of transition from CAPD+ICO to CAPD in year 2 | Beta | 0.0010 | 0.0001 | [1] |
| Probability of transition from CAPD+ICO to CAPD in year 3 onward | Beta | 0.0010 | 0.0001 | [1] |
| Probability of transition from CAPD+ICO to Death in year 1 | Beta | 0.1167 | 0.0117 | [1] |
| Probability of transition from CAPD+ICO to Death in year 2 | Beta | 0.1633 | 0.0163 | [1] |
| Probability of transition from CAPD+ICO to Death in year 3 onward | Beta | 0.1212 | 0.0121 | [1] |
| Probability of transition from CAPD+ICO to HD in year 1 | Beta | 0.0500 | 0.0050 | [1] |
| Probability of transition from CAPD+ICO to HD in year 2 | Beta | 0.1020 | 0.0102 | [1] |
| Probability of transition from CAPD+ICO to HD in year 3 onward | Beta | 0.2424 | 0.0242 | [1] |
| Probability of transition from CAPD+ICO to KT in year 1 | Beta | 0.0167 | 0.0017 | [1] |
| Probability of transition from CAPD+ICO to KT in year 2 | Beta | 0.0167 | 0.0017 | [1] |
| Probability of transition from CAPD+ICO to KT in year 3 onward | Beta | 0.0167 | 0.0017 | [1] |
| Probability of transition from HD to Death in year 1 | Beta | 0.26931 | 0.02693 | [2] |
| Probability of transition from HD to Death in year 2 | Beta | 0.12647 | 0.01265 | [2] |
| Probability of transition from HD to Death in year 3 | Beta | 0.08790 | 0.00879 | [2] |
| Probability of transition from HD to Death in year 4 | Beta | 0.08844 | 0.00884 | [2] |
| Probability of transition from HD to Death in year 5 | Beta | 0.06716 | 0.00672 | [2] |
| Probability of transition from HD to KT in year 1 | Beta | 0.00556 | 0.00056 | [1] |
| Probability of transition from HD to KT in year 2 onward | Beta | 0.00500 | 0.00050 | [1] |
| Probability of transition from KT to Death in year 1 | Beta | 0.03500 | 0.00350 | [3] |
| Probability of transition from KT to Death in year 2 | Beta | 0.01036 | 0.00104 | [3] |
| Probability of transition from KT to Death in year 3 | Beta | 0.00524 | 0.00052 | [3] |
| Probability of transition from KT to Death in year 4 onward | Beta | 0.01684 | 0.00168 | [3] |
| **Direct medical cost (DMC) in Thai baht (USD)** |  |  |  |  |
| ***Erythropoietin (EPO)*** |  |  |  |  |
| Cost of erythropoietin (per dose) | Gamma | 180 (5.179) | 18 (0.518) | [1] |
| Number of EPO doses for CAPD (per week) | Gamma | 1.700 | 0.099 | [1] |
| Number of EPO doses for CAPD+ICO (per week) | Gamma | 1.790 | 0.100 | [1] |
| Number of EPO doses for APD (per week) | Gamma | 1.770 | 0.111 | [1] |
| ***Solution*** |  |  |  |  |
| Cost of CAPD solution (baht/bag) | Gamma | 127 (3.654) | 12.72  (0.366) | [1] |
| Number of CAPD 1.5% solution (bags/week) | Gamma | 14.352 | 1.141 | [1] |
| Number of CAPD 2.5% solution (bags/week) | Gamma | 11.904 | 1.363 | [1] |
| Number of CAPD 4.25% solution (bags/week) | Gamma | 4.448 | 0.671 | [1] |
| Cost of Icodextrin (baht/bag) | Gamma | 450  (12.947) | 45  (1.295) | [1] |
| Number of Icodextrin (bags/week) | Gamma | 7.299 | 0.126 | [1] |
| Number of CAPD 1.5% solution (bags/week) | Gamma | 11.715 | 1.202 | [1] |
| Number of CAPD 2.5% solution (bags/week) | Gamma | 9.968 | 1.170 | [1] |
| Number of CAPD 4.25% solution (bags/week) | Gamma | 0.989 | 0.415 | [1] |
| Cost of APD solution (baht/bag) | Gamma | 298  (8.573) | 29.75  (0.856) | [1] |
| Number of APD 1.5% solution (bags/week) | Gamma | 9.967 | 0.792 | [1] |
| Number of APD 2.5% solution (bags/week) | Gamma | 8.014 | 0.772 | [1] |
| Number of APD 4.25% solution (bags/week) | Gamma | 1.207 | 0.431 | [1] |
| ***Inpatient department (IPD)*** |  |  |  |  |
| Number of IPD visits for CAPD (per year) | Gamma | 1.18 | 0.329 | [1] |
| IPD cost for CAPD in year 1 (per event) | Gamma | 65,398  (1,882) | 6,540  (188) | [1] |
| IPD cost for CAPD in year 2 (per event) | Gamma | 142,360  (4,096) | 14,236  (410) | [1] |
| IPD cost for CAPD in year 3 onward (per event) | Gamma | 48,871  (1,406) | 4,887  (141) | [1] |
| Number of IPD visits for CAPD+ICO (per year) | Gamma | 1.61 | 0.396 | [1] |
| IPD cost for CAPD+ICO in year 1 (per event) | Gamma | 36,022  (1,036) | 3,602  (104) | [1] |
| IPD cost for CAPD+ICO in year 2 (per event) | Gamma | 45,541  (1,310) | 4,554  (131) | [1] |
| IPD cost for CAPD+ICO in year 3 onward (per event) | Gamma | 60,359  (1,737) | 6,036  (174) | [1] |
| Number of IPD visits for APD (per year) | Gamma | 1.96 | 0.560 | [1] |
| IPD cost for APD in year 1 (per event) | Gamma | 72,729  (2,092) | 7,273  (209) | [1] |
| IPD cost for APD in year 2 (per event) | Gamma | 67,201  (1,933) | 6,720  (193) | [1] |
| IPD cost for APD in year 3 onward (per event) | Gamma | 76,575  (2,203) | 7,657  (220) | [1] |
| ***Outpatient department (OPD)*** |  |  |  |  |
| OPD cost for CAPD in year 1 (per year) | Gamma | 8,265  (238) | 826  (24) | [1] |
| OPD cost for CAPD in year 2 (per year) | Gamma | 6,190  (178) | 619 (18) | [1] |
| OPD cost for CAPD in year 3 onward (per year) | Gamma | 6,881  (198) | 688  (20) | [1] |
| OPD cost for CAPD+ICO in year 1 (per year) | Gamma | 8,555  (246) | 855  (25) | [1] |
| OPD cost for CAPD+ICO in year 2 (per year) | Gamma | 9,002  (259) | 900  (26) | [1] |
| OPD cost for CAPD+ICO in year 3 onward (per year) | Gamma | 9,724  (280) | 972  (28) | [1] |
| OPD cost for APD in year 1 (per year) | Gamma | 7,123  (205) | 712  (20) | [1] |
| OPD cost for APD in year 2 (per year) | Gamma | 5,284  (152) | 528  (15) | [1] |
| OPD cost for APD in year 3 onward (per year) | Gamma | 4,733  (136) | 473  (14) | [1] |
| Cost of switching therapy methods to HD | Gamma | 57,565  (1,656) | 13,015  (374) | [1] |
| IPD cost for HD (per year) | Gamma | 55,771  (1,605) | 7,656  (220) | [4] |
| OPD cost for HD (per week) | Gamma | 8,443  (243) | 2,595  (75) | [1] |
| Cost of switching therapy methods to KT | Gamma | 355,579  (10,230) | 79,717  (2,293) | [2] |
| OPD cost for KT in year 1 (per year) | Gamma | 359,927  (10,355) | 7,139  (205) | [2] |
| OPD cost for KT year 2 onward (per year) | Gamma | 257,736  (7,415) | 6,551  (188) | [2] |
| **Direct non-medical costs (DNMC)** |  |  |  |  |
| ***Outpatient department (OPD)*** |  |  |  |  |
| Travel cost per OPD visit | Gamma | 603  (17) | 68  (2) | [1] |
| Additional food cost per OPD visit | Gamma | 201  (6) | 10  (0.288) | [1] |
| Accommodation cost per OPD visit | Gamma | 1.299  (0.037) | 1.299  (0.037) | [1] |
| Caregiver cost per OPD visit | Gamma | 81  (2) | 17  (0.489) | [1] |
| Number of OPD visit for CAPD (per year) | Gamma | 15.46 | 2.215 | [1] |
| Number of OPD visit for CAPD+ICO (per year) | Gamma | 14.85 | 2.111 | [1] |
| Number of OPD visit for APD (per year) | Gamma | 15.67 | 1.600 | [1] |
| ***Inpatient department (IPD)*** |  |  |  |  |
| Travel cost per IPD visit | Gamma | 600  (17) | 106  (3) | [1] |
| Additional food cost per IPD visit | Gamma | 391  (11) | 64  (2) | [1] |
| Accommodation cost per IPD visit | Gamma | 124  (4) | 119  (3) | [1] |
| Caregiver cost per IPD visit | Gamma | 1,342  (39) | 434  (12) | [1] |
| Number of IPD visits for CAPD (per year) | Gamma | 1.1807 | 0.32853 | [1] |
| The number of IPD visits for CAPD+ICO (per year) | Gamma | 1.6067 | 0.39600 | [1] |
| The number of IPD visits for APD (per year) | Gamma | 1.9607 | 0.55971 | [1] |
| ***Caregiver, other treatment, and home renovation*** |  |  |  |  |
| Cost of informal caregiver for daily life (per year) | Gamma | 167,864  (4,829) | 13,009  (374) | [1] |
| Cost of formal caregivers (per month) | Gamma | 3,510  (101) | 1,910  (55) | [1] |
| Cost of treatment outside hospital (per year) | Gamma | 205  (6) | 92  (3) | [1] |
| Cost of buying accommodation (one time) | Gamma | 4,911  (141) | 511  (15) | [1] |
| Cost of home renovation (one time) | Gamma | 11,179  (322) | 1,552  (45) | [1] |
| DMNC for HD in year 1 onward (per year) | Gamma | 63,123  (1,816) | 14,525  (418) | [5] |
| DMNC for KT in year 1 onward (per year) | Gamma | 33,546  (965) | 8,141  (234) | [5] |
| **Utility** |  |  |  |  |
| Utility of CAPD | Beta | 0.790 | 0.017 | [1] |
| Utility of CAPD with AE | Beta | 0.620 | 0.109 | [1] |
| Utility of CAPD without AE | Beta | 0.74 | 0.016 | [1] |
| Utility of CAPD+ICO with AE | Beta | 0.630 | 0.119 | [1] |
| Utility of CAPD+ICO without AE | Beta | 0.82 | 0.013 | [1] |
| Utility of APD with AE | Beta | 0.543 | 0.175 | [1] |
| Utility of APD without AE | Beta | 0.81 | 0.013 | [1] |
| Utility of HD | Beta | 0.753 | 0.020 | [6] |
| Utility of KT | Beta | 0.826 | 0.024 | [7] |

**CAPD indicates continuous ambulatory peritoneal dialysis; APD, automated peritoneal dialysis; HD, hemodialysis; KT, kidney transplantation; IPD, inpatient department; OPD, outpatient department; ICO, icodextrin; PD, peritoneal dialysis; AE, adverse event; SE, standard error; EPO, erythropoietin, DMC, direct medical costs; DMNC, direct non-medical costs; USD, United States dollar; 2023 exchange rate of 1 Thai baht equal to 0.02877 USD**

**References**

1. Sritippayawan S, Chaikledkaew U, Thavorncharoensap M, Youngkong S, Chuengsaman P, Tongsai S, et al. Feasibility study of the inclusion of automated peritonieal dialysis and icodextrin solution for fluid overload adult chronic ESKD patients in the National Health Security System of Thailand. Nonthaburi: Health Systems Research Institute; 2024.

2. The Nephrology Society of Thailand. Annual Report Thailand Renal Replacement Therapy 2007-2022. Bangkok: The Nephrology Society of Thailand.

3. Thai Transplant Society. Kidney transplantation registry in Thailand. Bangkok: Thai Transplant Society. .

4. Security OoNH. Office of National Health Security. Meeting to Clarify Criteria, Methods, and Conditions for Reimbursement of Expenses for Patients with Chronic Kidney Disease in the National Health Security System for the Fiscal In year 2022. 2 Feb. 2022. 2022.

5. HITAP Ministry of Public Health. Standard Cost Lists for Health Technology Assessment Thailand: HITAP Ministry of Public Health 2010 [Available from: <https://costingmenu.hitap.net/>.

6. Thaweethamcharoen T, Sakulbumrungsil R, Nopmaneejumruslers C, Vasuvattakul S. Cost-utility analysis of erythropoietin for anemia treatment in thai end-stage renal disease patients with hemodialysis. Value in Health Regional Issues. 2014;3:44-9.

7. Li B, Cairns JA, Draper H, Dudley C, Forsythe JL, Johnson RJ, et al. Estimating health-state utility values in kidney transplant recipients and waiting-list patients using the EQ-5D-5L. Value in Health. 2017;20(7):976-84.
