# Supplementary material for: Economic evaluation of dialysis treatment in end-stage renal disease patients with fluid and sodium overload: Evidence from a randomized controlled trial in Thailand
Source: PLoS One. 2025 Nov 5;20(11):e0335749. doi: 10.1371/journal.pone.0335749 (PMC12588515; doi:10.1371/journal.pone.0335749)
Supplement: S3 Table — (DOCX) [file pone.0335749.s003.docx]

**Table S3** **Scenario Analysis Results for a Change in the Probability of Death from Year 3 Onward.**

| **% Increase in probability of death in year 3 onward** | **CAPD+ICO vs. CAPD** | | | | | **APD vs. CAPD** | | | | |
| --- | --- | --- | --- | --- | --- | --- | --- | --- | --- | --- |
|  | **Incremental** | | | **ICER** | | **Incremental** | | | **ICER** | |
|  | **Cost**  **(USD)** | **LY** | **QALY** | **Cost per LY gained**  **(USD)** | **Cost per QALY gained**  **(USD)** | **Cost**  **(USD)** | **LY** | **QALY** | **Cost per LY gained**  **(USD)** | **Cost per QALY gained**  **(USD)** |
| 0% | 942,816  (27,125) | 1.36 | 1.04 | 692,532  (19,924) | 908,440  (26,136) | 136,241  (3,920) | -0.14 | -0.22 | -956,969  (-27,532) | -619,738  (-17,830) |
| 10% | 954,445  (27,459) | 1.39 | 1.06 | 686,129  (19,740) | 903,617  (25,997) | 149,509  (4,301) | -0.11 | -0.18 | -1,343,495  (-38,652) | -848,645  (-24,416) |
| 20% | 963,284  (27,714) | 1.42 | 1.07 | 680,671  (19,583) | 899,543  (25,880) | 161,157  (4,636) | -0.08 | -0.14 | -1,928,315  (-55,478) | -1,182,966  (-34,034) |
| 30% | 969,768  (27,900) | 1.43 | 1.08 | 675,963  (19,447) | 896,071  (25,780) | 171,415  (4,932) | -0.06 | -0.10 | -2,915,879  (-83,890) | -1,721,495  (-49,527) |
| 40% | 974,262  (28,030) | 1.45 | 1.09 | 671,859  (19,329) | 893,091  (25,694) | 180,473  (5,192) | -0.04 | -0.07 | -4,938,975  (-142,094) | -2,741,696  (-78,879) |
| 50% | 977,070  (28,110) | 1.46 | 1.10 | 668,250  (19,226) | 890,517  (25,620) | 188,491  (5,423) | -0.02 | -0.03 | -11,414,297  (-328,389) | -5,437,264  (-156,430) |
| 60% | 978,449  (28,150) | 1.47 | 1.10 | 665,051  (19,134) | 888,282  (25,556) | 195,606  (5,628) | 0.00 | -0.01 | 124,784,331  (3,590,045) | -33,604,092  (-966,790) |
| 70% | 978,614  (25,155) | 1.48 | 1.10 | 662,195  (19,051) | 886,334  (25,500) | 201,932  (5,810) | 0.02 | 0.02 | 11,258,237  (323,899) | 9,638,253  (227,293) |
| 80% | 977,750  (28,130) | 1.48 | 1.11 | 659,631  (18,978) | 884,630  (25,451) | 207,567  (5,972) | 0.03 | 0.05 | 6,329,634  (182,104) | 4,526,349  (130,223) |
| 90% | 976,011  (28,080) | 1.48 | 1.11 | 657,314  (18,911) | 883,137  (25,408) | 212,596  (6,116) | 0.05 | 0.07 | 4,590,766  (132,076) | 3,077,521  (88,540) |
| 100% | 973,531  (28,008) | 1.49 | 1.10 | 655,212  (18,850) | 881,825  (25,370) | 217,091  (6,246) | 0.06 | 0.09 | 3,702,410  (106,518) | 2,391,425  (68,801) |

CAPD indicates continuous ambulatory peritoneal dialysis; APD, automated peritoneal dialysis; ICO, icodextrin; LY, life years; QALY, quality-

adjusted life years; ICER, incremental cost-effectiveness ratio; USD, United States dollar
