# Supplementary material for: Economic evaluation of dialysis treatment in end-stage renal disease patients with fluid and sodium overload: Evidence from a randomized controlled trial in Thailand
Source: PLoS One. 2025 Nov 5;20(11):e0335749. doi: 10.1371/journal.pone.0335749 (PMC12588515; doi:10.1371/journal.pone.0335749)
Supplement: S4 Table — (DOCX) [file pone.0335749.s004.docx]

**Table S4 Scenario Analysis Results for a Change in Mortality Rates and Health Utility Values Based on Yang et al.’s study in Singapore.**

| **Results in baht**  **(USD)** | **CAPD** | **CAPD+ICO** | **APD** |
| --- | --- | --- | --- |
| **Total cost** | 2,487,161  (71,556) | 3,429,977  (98,680) | 2,623,402  (75,475) |
| **Total LYs** | 4.95 | 5.12 | 5.13 |
| **Total QALYs** | 3.81 | 3.95 | 3.33 |
| **Incremental costs** |  | 942,816  (27,125) | 136,241  (3,920) |
| **Incremental LYs** |  | 0.17 | 0.18 |
| **Incremental QALYs** |  | 0.14 | -0.48 |
| **ICER per LY gained** |  | 5,545,976  (159,559) | 756,894  (21,778) |
| **ICER per QALY gained** |  | 6,734,400  (193,753) | Dominated^a^ (-283,835)  (-8,157) |

^a^Dominated in this context refers to higher costs but lower LYs or QALYs. LYs, life years; QALY, quality adjusted life years; ICER, incremental cost-effectiveness ratio; USD, United States dollar
