# Supplementary material for: Economic evaluation of dialysis treatment in end-stage renal disease patients with fluid and sodium overload: Evidence from a randomized controlled trial in Thailand
Source: PLoS One. 2025 Nov 5;20(11):e0335749. doi: 10.1371/journal.pone.0335749 (PMC12588515; doi:10.1371/journal.pone.0335749)
Supplement: Attachment — Submitted filename: Responses to Reviewers-19May25.docx [file pone.0335749.s006.docx]

**Responses to Editors and Reviewers**

**Journal Requirements:**

When submitting your revision, we need you to address these additional requirements.

1. Please ensure that your manuscript meets PLOS ONE's style requirements, including those for file naming. The PLOS ONE style templates can be found at https://journals.plos.org/plosone/s/file?id=wjVg/PLOSOne_formatting_sample_main_body.pdf and https://journals.plos.org/plosone/s/file?id=ba62/PLOSOne_formatting_sample_title_authors_affiliations.pdf

Response: Thank you very much. We have ensured that our manuscript meets PLOS ONE's style requirements, including those for file naming.

2. Thank you for stating the following financial disclosure:

This research project has been funded by the Health Systems Research Institute. The findings, interpretations and conclusions expressed in this article do not necessarily reflect the views of the aforementioned funding agency. Please state what role the funders took in the study. If the funders had no role, please state: "The funders had no role in study design, data collection and analysis, decision to publish, or preparation of the manuscript."

If this statement is not correct you must amend it as needed. Please include this amended Role of Funder statement in your cover letter; we will change the online submission form on your behalf.

Response: Thank you very much. We have included this amended Role of Funder statement in our cover letter as follows: "The funders had no role in study design, data collection and analysis, decision to publish, or preparation of the manuscript."

3. Please remove all personal information, ensure that the data shared are in accordance with participant consent, and re-upload a fully anonymized data set. Note: spreadsheet columns with personal information must be removed and not hidden as all hidden columns will appear in the published file. Additional guidance on preparing raw data for publication can be found in our Data Policy (https://journals.plos.org/plosone/s/data-availability#loc-human-research-participant-data-and-other-sensitive-data) and in the following article: http://www.bmj.com/content/340/bmj.c181.long.

Response: Thank you for your suggestion. Our study involved end-stage renal disease patients with fluid and sodium overload, and the dataset contains sensitive personal and clinical information. Due to ethical restrictions and participant confidentiality concerns, we are unable to publicly share the full dataset, even in anonymized form, as per the conditions of participant consent and institutional review board approval. However, data are available upon reasonable request to qualified researchers, subject to institutional review and approval. Requests may be directed to the Human Research Protection Unit, Faculty of Medicine Siriraj Hospital, Mahidol University, at Room 210, 2nd Floor, His Majesty the King's 80th Birthday Anniversary 5th December 2007 Building, 2 Wang Lang Road, Bangkoknoi, Bangkok 10700, or via email at [siethics@mahidol.ac.th](mailto:siethics@mahidol.ac.th).

4. Please include captions for your Supporting Information files at the end of your manuscript, and update any in-text citations to match accordingly. Please see our Supporting Information guidelines for more information: http://journals.plos.org/plosone/s/supporting-information.

Response: Thank you very much. We have include captions for our Supporting Information files at the end of our manuscript.

**Additional Editor Comments:**

Thank you for considering PLOS One for the publication of your valuable article. This manuscript is well-conducted and yields important information. However, there are some comments from the reviewers that require your attention. Furthermore, I would appreciate clarification regarding reference number 12. Specifically, I would like to inquire whether this reference has undergone peer review. Although the reference is in Thai, it contains tables and figures that exhibit similarities to those presented in your submitted manuscript.

Response: Thank you for your comment regarding reference number 12. We would like to clarify that reference 12 is a Thai-language research report, produced as part of a project conducted by Mahidol University and submitted to the Health Systems Research Institute. The report has not undergone peer review and was intended for internal or policy use. It is not indexed or published in any academic journal. While the report includes similar tables and figures, the manuscript we submitted has been substantially revised, reorganized, and rewritten to meet academic journal standards. The analysis in the manuscript also incorporates updated methodologies and English-language presentation tailored to an international audience. We have cited the report in accordance with transparency and ethical standards. Furthermore, we disclosed the existence of this earlier report in our cover letter and confirmed that the submitted manuscript represents an original and unpublished academic work. Please let us know if any further clarification or revision is required.

**Reviewers' comments:**

**Reviewer #1:**

1.The study employs a Markov model to evaluate the cost-utility of CAPD+ICO compared to glucose-based CAPD and APD in ESRD patients in Thailand. While the study appears well-conducted, several issues should be addressed before publication. First, the study relies heavily on data from a randomized controlled trial (RCT) conducted across 16 hospitals in Thailand. Although the use of RCT data is a strength, the authors provide insufficient detail on the inclusion and exclusion criteria for the study population. Clarifying whether the sample includes only dialysis patients with fluid and sodium overload or the general dialysis population is crucial for interpreting the results.

Response: Thank you very much for your insightful comment. We agree with your suggestion and have revised the manuscript accordingly. We have added more detailed information on the inclusion and exclusion criteria as well as treatment regimens. Additionally, we have clarified that the sample consisted exclusively ESRD patients with fluid and sodium overload as follows.

*(Model parameters, line 123-164, page 6-8).*

*“The RCT study employed a two-stage randomization process to minimize bias and ensure comparability across treatment groups. Block randomization was used within each participating hospital to balance treatment allocation, while stratified randomization accounted for key prognostic factors, specifically duration of PD treatment (≤3 years vs. >3 years) and the type of APD machine used (Baxter vs. Fresenius Medical Care).*

*Eligible participants were adults aged 18 years or older enrolled in Thailand’s UHC scheme and had been receiving CAPD for more than three months at Ministry of Public Health or public university hospitals across various regions of Thailand. Inclusion criteria required patients to use 2-liter per cycle PD solutions for three to five cycles daily, including at least two cycles with 2.5% dextrose or at least one cycle with 4.25% dextrose. Additionally, participants had to exhibit at least one clinical indicator of fluid overload i.e., edema, poorly controlled hypertension (systolic blood pressure (SBP) >140 mmHg or diastolic blood pressure (DBP) >90 mmHg), or a history of congestive heart failure symptoms (CHF) symptoms within the past year. Patients also had to be able to communicate in Thai to complete health-related quality of life (HRQoL) assessments.*

*Exclusion criteria included edema from non-fluid-related causes such as catheter malfunction (e.g., malposition or omental wrapping) or suspected dialysate leakage confirmed by a rapid fill-and-drain test (discrepancy ≥ 200 mL between infused and drained volumes). Patients were also excluded for edema due to medication side effects, hypothyroidism, liver cirrhosis, or malnutrition (defined as serum albumin <2 g/dL within the past two months). Additional exclusions included peritonitis within the previous month, baseline SBP <100 mmHg, current diagnosis of sepsis, acute coronary syndrome, CHF, or stroke, prior use of icodextrin or home-based APD, pregnancy, terminal cancer or life expectancy less than six months, or any change in healthcare coverage during the study period.*

*Patients were randomized to one of three PD modalities i.e., CAPD, CAPD+ICO, or APD. All patients received monthly treatment adjustments aimed at resolving fluid and sodium overload defined as resolution of edema, stable BP (100–140/<90 mmHg) without dizziness, and absence of CHF symptoms, and achieving adequate solute clearance (total weekly Kt/V urea >1.7 or normalized creatinine clearance >45 L/1.73 m²) within two months. CAPD patients had their number of exchanges and glucose concentrations adjusted as needed. In the CAPD+ICO group, one low-glucose exchange was replaced with icodextrin, with similar adjustments to remaining exchanges. APD patients started with a nighttime intermittent PD regimen using 10 liters of 2.5% dextrose with adjustments up to 20 liters as necessary, and day dwell exchanges were added if fluid overload or inadequate clearance persisted. Tidal PD was permitted in cases of inflow/outflow discomfort or suboptimal drainage.*

*A total of 180 patients were enrolled between November 16, 2020 – March 8, 2021, and were equally allocated into CAPD, CAPD+ICO, and APD groups (60 patients each) [12]. Participants were recruited from 16 hospitals across all regions of Thailand, with most hospitals. enrolling 12 patients (4 per group), except for two that enrolled 6 patients each. Patients were followed up every two months to assess their clinical outcomes and determine transitional probabilities, including death, changes in dialysis modality, or transition to KT, over a three-year period [12].”*

2. Second, it is unclear whether the RCT was designed to inform all model inputs. Notably, significant differences in mortality and dialysis modality switching rates were observed among the three patient groups—parameters that are central to the cost-effectiveness estimates. If the RCT does not provide reliable estimates for these inputs and the study aims to inform national policy, the authors should supplement their analysis with evidence from additional studies to assess the consistency of these findings. Without this, the generalizability and policy relevance of the results are uncertain.

Response: Thank you for your valuable comment and we apologize for not making this point clearer in the initial manuscript. The RCT was specifically designed to inform all key model inputs required for the economic evaluation of CAPD+ICO and APD compared to glucose-based CAPD over a lifetime horizon, with the aim of supporting national dialysis policy decisions. Most of the model input parameters such as transition probabilities, costs, and utility values were derived directly from the RCT data collected during the three-year RCT. We have clarified this point on the manuscript in the Methods and Discussion sections as follows.

(*Study design, lines 98-100, page 5)*

*“Data, including transitional probabilities, direct medical and non-medical costs, and utilities, were specifically collected from a randomized controlled trial (RCT) conducted across 16 hospitals in various regions of Thailand [12].”*

*(Discussion, lines 315-319, page 16)*

*“Our study is the first study to conduct a cost-utility analysis of CAPD+ICO and APD compared with glucose-based CAPD using data on transitional probabilities, costs and utility values from a previous RCT study [12]. The RCT was specifically designed to generate key model inputs required for this study aiming to inform policy decision making toward dialysis modality selection.”*

*(Discussion, lines 424-427 page 20)*

*“The strengths of this study included the use of data from a national RCT [12] designed specifically to inform long-term economic evaluation of CAPD+ICO and APD versus CAPD with glucose. Most key inputs i.e., transition probabilities, costs, and utility values were derived directly from the trial, improving internal validity.”*

*In addition, we compared our findings with previously published studies conducted in Thailand and found that our results were consistent with those studies. This consistency enhances both the generalizability and policy relevance of our findings. We have revised the manuscript to clarify this point in the Discussion section as follows.*

*(Discussion, lines 329-351, page 16-17)*

*“Moreover, patients in the APD group experienced a significantly greater decline in EQ-5D-5L scores over time compared to those in CAPD and CAPD+ICO groups, aligning with clinical findings that suggest poorer outcomes among APD patients in this context. While APD has been associated with greater convenience and improved HRQoL in high-income settings, evidence from other contexts has shown more variable results. For example, a prospective cohort study from the Netherlands, using data from the Netherlands Cooperative Study on the Adequacy of Dialysis (NECOSAD), found no statistically significant differences in HRQoL between patients receiving APD and those on CAPD over a three-year follow-up period [16]. Similarly, a study from Singapore reported only marginal improvements in specific physical health domains among APD patients, with no significant differences in overall HRQoL compared to CAPD [17]. These findings suggest that the benefits of APD may not be universally experienced. Variability in patient characteristics, availability of caregiver support, health system infrastructure, and dialysis practices may influence patient-reported outcomes. In resource-limited settings such as Thailand, the practical advantages of APD may be diminished if patients rely heavily on family members for machine setup, monitoring, and troubleshooting. This dependence can reduce the perceived convenience and potentially impact quality of life. Although the current NHSO policy promotes APD as the preferred modality, our results demonstrate its relatively reduced cost-effectiveness. These findings are consistent with previous research conducted at Siriraj Hospital, which also found that APD was associated with higher lifetime costs and lower QALYs when compared to CAPD using glucose solution [18, 19]. However, the observed differences in cost and utility outcomes reflect the real-world implementation of APD in the Thai setting, where some expected advantages may not be fully realized or adequately captured. Therefore, future studies should explore on other potential benefits of APD within the Thai context.”*

3. Third, the model lacks validation, which is particularly important given the relatively modest life years gained (LY) across scenarios. This may, in part, stem from the assumption of a constant and equal mortality rate beyond year three, which warrants a more robust approach. A thorough sensitivity analysis or alternative assumptions would help strengthen the credibility and applicability of the model's results.

Response: Thank you very much for your insightful suggestions. We appreciate the reviewer’s concern regarding model validation and the assumptions applied beyond the third year. As our RCT data only covered the first three years, we acknowledge the limitation in long-term mortality data, particularly for the specific population of ESRD patients with fluid and sodium overload. To address this, we assumed a constant mortality rate beyond year 3 based on the average annual mortality observed in years 1–3 in our RCT. While this approach is common in the absence of long-term data, we agree it may underestimate variability in long-term outcomes. To enhance the model's credibility, the structure and input parameters were reviewed for face validity by a panel of clinical experts and health economists to ensure appropriateness and relevance to the Thai context. Additionally, we performed extensive sensitivity analyses including one-way and probabilistic sensitivity analyses to test the effect of mortality and other key parameters. Furthermore, scenario analysis in which the probability of death in year 3 was varied from 0% to 100% increase to test the impact of uncertainty around mortality parameter. These analyses demonstrated that although ICER values varied, the overall cost-effectiveness results of interventions remained consistent in Table S2.

*(Economic model, lines 110-112, page 6)*

*“The model structure and input parameters underwent face validity assessment by a panel of clinical experts and health economists to ensure appropriateness and relevance to the Thai context.”*

*(Uncertainty analysis, lines 217-218, page 11)*

*“To account for the uncertainty of various input variables, one-way sensitivity and probabilistic sensitivity analyses (PSA)as well as scenario analysis were conducted.”*

*(Uncertainty analysis, lines 222-223, page 11)*

*“Furthermore, scenario analysis was conducted to explore the effect of key parameters that significantly impacted ICER values.”*

*(Scenario analysis, lines 265-284, page 13-14)*

*“As the RCT data only covered the first three years, we assumed a constant mortality rate beyond year 3 based on the average annual mortality observed in years 1–3. A scenario analysis was conducted to assess the impact of increasing the probability of death from year 3 onward (ranging from 0% to 100%) on the ICER values of CAPD+ICO and APD compared to glucose-based CAPD (Table S2). For CAPD+ICO, both LYs and QALYs gained increased slightly across all scenarios, while the incremental cost remained relatively stable. The ICER values ranged from 908,440 baht (26,082 USD) per QALY gained at 0% increase in mortality to 881,825 baht (25,370 USD) per QALY gained at a 100% increase, indicating a modest improvement in cost-effectiveness as mortality assumptions became more conservative. The intervention remained consistently not cost-effective across all mortality scenarios at the societal WTP of 160,000 baht (4,603 USD) per QALY gained.*

*For APD, the intervention was associated with negative incremental QALYs up to a 50% increase in mortality, reflecting inferior effectiveness compared to CAPD with glucose under those assumptions. At 60% and beyond, incremental QALYs turned positive, but the ICER values were substantially higher, ranging from 9.6 to over 33 million baht per QALY gained. This indicates that APD remained not cost-effective under most scenarios at the current WTP threshold. Overall, the scenario analysis demonstrated that the relative cost-effectiveness ranking of the three dialysis modalities remained stable across a wide range of long-term mortality assumptions, supporting the robustness of the model outcomes.”*

*We have revised the manuscript to clarify this modeling assumption, present uncertainty analysis results and justify our approach. The limitations have also been explicitly discussed. We agree that future research with longer-term follow-up or real-world cohort data would be valuable for refining long-term survival estimates in this population.*

*(Discussion, lines 448-457, page 21-22)*

*“Lastly, the long-term mortality assumptions beyond year three were based on data from our RCT with a follow-up period limited to three years. Due to the lack of long-term data, particularly for ESRD patients with fluid and sodium overload, we assumed a constant mortality rate beyond year three, based on the average annual mortality observed during the trial period. While this assumption is commonly applied in health economic models, it may not fully capture the variability in long-term survival outcomes. Although extensive sensitivity analyses including one-way, probabilistic, and scenario analyses varying the probability of death were performed to test the robustness of this assumption, the results should be interpreted with caution. Future studies incorporating longer-term follow-up data or real-world evidence are warranted to improve the accuracy of survival projections and enhance model validity.”*

4. Fourth, there is a disconnect between the study’s findings and its policy recommendations. While the analysis suggests that CAPD+ICO does not represent good value for money, the authors recommend its inclusion in the benefit package based on clinical benefits and financial protection. While these factors are important for policymakers, they extend beyond the typical scope of a cost-utility analysis and may confuse readers about the study’s primary conclusions.

Response: Thank you very much for great suggestions. We agree that there is disconnect between the study’s findings and its policy recommendations. We have revised sentences related to policy recommendations in the Abstract, Discussion and Conclusion sections as follows.

*(Abstract, lines 30-39, page 2)*

*“Compared to glucose-based CAPD, the incremental cost-effectiveness ratio (ICER) for CAPD+ICO was 908,440 THB (26,082 USD) per quality-adjusted life year (QALY) gained, while APD was dominated, incurring higher costs and yielding fewer QALYs. The results indicated that glucose-based CAPD had a 90% probability of being the most cost-effective option from a societal perspective, based on Thailand’s willingness-to-pay threshold of 160,000 THB (4,603 USD) per QALY gained. Therefore, CAPD+ICO is not considered a good value for money, requiring an additional annual budget of approximately 58 million THB (2 million USD). These findings provide important economic evaluation evidence to support policy decision-making alongside clinical effectiveness and equity considerations in guiding future UHC benefit package decisions for dialysis modalities among ESRD patients with fluid and sodium overload in Thailand.”*

*(Discussion, lines 392-400, page 19).*

*“From a purely cost-effectiveness standpoint, our findings indicate that at a WTP threshold of 160,000 baht (4,603 USD) per QALY gained, CAPD+ICO is not considered cost-effective when compared to CAPD using glucose solution, resulting in an average additional budget requirement of approximately 58 million baht (2 million USD) per year. However, it is important to note that patients treated with CAPD+ICO demonstrated improved clinical outcomes, including longer life expectancy and better HRQoL [12]. Although these clinical benefits extend beyond the conventional focus of a cost-utility analysis, they may be relevant to policymakers seeking to balance economic efficiency with considerations of health equity, clinical effectiveness, and patient-centered outcomes.”*

*(Conclusion, lines 459-466, page 22).*

*“At the Thai societal willingness-to-pay threshold of 160,000 baht (4,603 USD) per QALY gained, glucose-based CAPD had a 90% probability of being the most cost-effective option from a societal perspective. CAPD+ICO is not considered cost-effective, with an estimated additional budget impact of approximately 58 million baht (2 million USD) per year. APD was found to be a dominated option, offering lower QALYs at higher costs compared to glucose-based CAPD. The results of this study can serve as economic efficiency evidence to inform policy decisions, while also taking into account clinical outcomes and equity considerations in future UHC benefit package decisions on dialysis modalities for ESRD patients with fluid and sodium overload in Thailand.”*

5. Lastly, the recent implementation of the NHSO’s APD-first policy in 2024 may affect the study’s relevance and its policy implications. The authors should acknowledge and discuss this shift in the manuscript.

Response: Thank you very much for great suggestion. We have added this point as suggested in the Discussion section.

*(Discussion, lines 403-406, page 19-20)*

*“It is worth noting that the NHSO implemented an APD-first policy in 2024, which may affect the relevance and application of this study’s findings. However, this analysis remains valuable in informing future policy refinement, particularly for subpopulations such as ESRD patients with fluid and sodium overload.”*

**Reviewer #2:**

This manuscript presents a comprehensive cost-utility and budget impact analysis of peritoneal dialysis modalities (CAPD, CAPD+ICO, and APD) among Thai patients with ESRD and fluid/sodium overload. The topic is highly relevant and timely, given Thailand’s evolving dialysis policy and the ongoing burden of ESRD. The study is methodologically sound, uses primary data from a multi-center RCT, and adheres to standard HTA practices. The findings are well-structured and could provide valuable insights for policymakers. However, several areas require clarification or strengthening to enhance the manuscript’s scientific rigor, clarity, and policy relevance:

1. Please provide more detail on the patients' clinical conditions and treatment regimens across the three groups, as the cited RCT report is in Thai. This would help international readers better understand the trial procedures.

Response: Thank you very much for your insightful comment. We agree with your suggestion and have revised the manuscript accordingly. We have added more detailed information on the inclusion and exclusion criteria as well as treatment regimens. Additionally, we have clarified that the sample consisted exclusively ESRD patients with fluid and sodium overload as follows.

*(Model parameters, line 123-164, page 6-8).*

*“The RCT study employed a two-stage randomization process to minimize bias and ensure comparability across treatment groups. Block randomization was used within each participating hospital to balance treatment allocation, while stratified randomization accounted for key prognostic factors, specifically duration of PD treatment (≤3 years vs. >3 years) and the type of APD machine used (Baxter vs. Fresenius Medical Care).*

*Eligible participants were adults aged 18 years or older enrolled in Thailand’s UHC scheme and had been receiving CAPD for more than three months at Ministry of Public Health or public university hospitals across various regions of Thailand. Inclusion criteria required patients to use 2-liter per cycle PD solutions for three to five cycles daily, including at least two cycles with 2.5% dextrose or at least one cycle with 4.25% dextrose. Additionally, participants had to exhibit at least one clinical indicator of fluid overload i.e., edema, poorly controlled hypertension (systolic blood pressure (SBP) >140 mmHg or diastolic blood pressure (DBP) >90 mmHg), or a history of congestive heart failure symptoms (CHF) symptoms within the past year. Patients also had to be able to communicate in Thai to complete health-related quality of life (HRQoL) assessments.*

*Exclusion criteria included edema from non-fluid-related causes such as catheter malfunction (e.g., malposition or omental wrapping) or suspected dialysate leakage confirmed by a rapid fill-and-drain test (discrepancy ≥ 200 mL between infused and drained volumes). Patients were also excluded for edema due to medication side effects, hypothyroidism, liver cirrhosis, or malnutrition (defined as serum albumin <2 g/dL within the past two months). Additional exclusions included peritonitis within the previous month, baseline SBP <100 mmHg, current diagnosis of sepsis, acute coronary syndrome, CHF, or stroke, prior use of icodextrin or home-based APD, pregnancy, terminal cancer or life expectancy less than six months, or any change in healthcare coverage during the study period.*

*Patients were randomized to one of three PD modalities i.e., CAPD, CAPD+ICO, or APD. All patients received monthly treatment adjustments aimed at resolving fluid and sodium overload defined as resolution of edema, stable BP (100–140/<90 mmHg) without dizziness, and absence of CHF symptoms, and achieving adequate solute clearance (total weekly Kt/V urea >1.7 or normalized creatinine clearance >45 L/1.73 m²) within two months. CAPD patients had their number of exchanges and glucose concentrations adjusted as needed. In the CAPD+ICO group, one low-glucose exchange was replaced with icodextrin, with similar adjustments to remaining exchanges. APD patients started with a nighttime intermittent PD regimen using 10 liters of 2.5% dextrose with adjustments up to 20 liters as necessary, and day dwell exchanges were added if fluid overload or inadequate clearance persisted. Tidal PD was permitted in cases of inflow/outflow discomfort or suboptimal drainage.*

*A total of 180 patients were enrolled between November 16, 2020 – March 8, 2021, and were equally allocated into CAPD, CAPD+ICO, and APD groups (60 patients each) [12]. Participants were recruited from 16 hospitals across all regions of Thailand, with most hospitals. enrolling 12 patients (4 per group), except for two that enrolled 6 patients each. Patients were followed up every two months to assess their clinical outcomes and determine transitional probabilities, including death, changes in dialysis modality, or transition to KT, over a three-year period [12].”*

2. In the trial report, there was a substantial number of dropouts in the third year, which may increase the uncertainty of the model parameters for that period. This limitation should be discussed.

Response: Thank you very much for your valuable suggestion. We agree that the substantial number of dropouts in the third year may increase the uncertainty of the model parameters. We have addressed this limitation in the Discussion section as follows.

*(Discussion, lines 443-448, page 21)*

*“Fourthly, according to the RCT, there was a substantial number of dropouts in the third year, primarily due to death (45%) and transition to hemodialysis (22%), which may contribute to increased uncertainty in the model parameters for that period. The reduced number of participants during the third year of follow-up may limit the robustness of the clinical and economic outcomes derived from this time frame, thereby affecting the precision of transition probabilities and cost estimates used in the model.”*

3.From Table S1:o There are transitional probabilities listed from KT to PD/HD, which are inconsistent with Figure 1 and the explanation in line 130 (Methods: Economic Model). Please clarify or revise.

Response: Thank you very much for pointing out the inconsistency. We apologized for the typographical error in Table S1. Upon careful review, we confirm that the transitional probabilities from KT to PD/HD were not used in our Markov model. Therefore, we have removed these probabilities from Table S1. Only the probabilities of remaining in the KT state or transitioning from KT to death were included as model input parameters. To improve clarity, we have also revised Figure 1 accordingly.

4.The cost of inpatient (IPD) treatment during the second year per event in the CAPD group appears disproportionately high compared to other IPD costs. Please explain this anomaly.

Response: The disproportionately high inpatient (IPD) cost per event in the CAPD group during the second year was primarily driven by a single outlier case at one study site, where a patient was admitted to the intensive care unit (ICU) and subsequently passed away. This patient had an extended length of stay (LOS) of 63 days, which significantly increased the average cost for that group during the period. To evaluate the impact of this outlier, we conducted sensitivity analyses. Although the incremental cost-effectiveness ratio (ICER) was sensitive to variations in inpatient costs, the relative cost-effectiveness ranking of the treatment options remained unchanged, demonstrating the robustness of the model’s conclusions. We have added further clarification in the Discussion section as follows.

*(Discussion, lines 355-362, page 17-18)*

*“Furthermore, the disproportionately high inpatient cost per event in the CAPD group during the second year was primarily driven by a single outlier case at one study site, where a patient was admitted to the intensive care unit and subsequently passed away. This patient had an extended length of stay (LOS) of 63 days, which significantly increased the average cost for that group during the period. To evaluate the impact of this outlier, we conducted sensitivity analyses. Although the incremental cost-effectiveness ratio (ICER) was sensitive to variations in inpatient costs, the relative cost-effectiveness ranking of the treatment options remained unchanged, demonstrating the robustness of the model’s conclusions.”*

5. The rate of HD transfer from the CAPD+ICO group was significantly higher than from the CAPD group. Since this influences the ICER and is highlighted in the Tornado diagram, please discuss potential reasons behind this finding.

Response: Thank you very much for raising this important point. We agree that the rate of transition to HD from the CAPD+ICO group was significantly higher than from the CAPD group. This difference affects the ICER and is illustrated in the Tornado diagram. We have addressed the potential reasons for this finding in Discussion section as follows.

*(Discussion, lines 363-379, page 18).*

*“In addition, the higher rate of transition to HD in the CAPD+ICO group, compared to the CAPD group, warrants consideration. All patients enrolled in the trial had fluid and sodium overload at baseline, representing a clinically complex and high-risk population [12]. Volume overload is a known predictor of adverse outcomes in PD patients, including increased risks of technique failure and mortality [16, 20]. Although icodextrin is intended to improve ultrafiltration, factors such as peritoneal membrane dysfunction, persistent fluid overload, or severe infections may still necessitate modality change. The RCT found no statistically significant differences in peritonitis rates or dialysis adequacy among the three treatment arms. However, infection-related deaths were slightly lower in the CAPD+ICO group (47%) compared to CAPD (57%) and APD (56%). Notably, the CAPD group exhibited higher mortality rates in year 1 (0.1667), year 2 (0.2449), and year 3 (0.2424) than the CAPD+ICO group (0.1167, 0.1633, and 0.1212) [12]. This suggests that patients in the CAPD+ICO group, experiencing lower mortality, may have survived long enough for complications to develop, increasing the likelihood of transitioning to HD. Indeed, the* *CAPD+ICO group showed higher HD transfer probabilities across all years (0.0500, 0.1020, and 0.2424) compared to the CAPD group (0.0167, 0.0408, and 0.1515). In some cases, the severity of fluid overload or inadequate response to PD may have prompted clinicians to select HD as a more immediate and controlled strategy for volume management [21].”*

6. It is unclear how the authors assumed that the direct non-medical costs and opportunity costs in the APD group were the same as in the CAPD group, particularly since APD typically requires less caregiving time. Please clarify or justify this assumption.

Response: Thank you very much for valuable comment, and we apologize for confusion. Direct non-medical costs and opportunity costs in the APD group were not the same as those in CAPD group. We found that the total direct non-medical costs in the APD group were actually slightly higher than those in the CAPD group (APD=244,979 baht vs. CAPD=242,874 baht in year 1) and APD=228,890 baht vs. CAPD=226,785 baht in year 2). These costs include travel, food, accommodation, and caregiver expenses related to both OPD and IPD visits. The higher number of OPD and IPD visits in the APD group contributed to this difference. Please see revised Table S1 for more details on cost items.

While APD may reduce caregiving burden in some contexts due to its overnight operation and reduced frequency of daytime exchanges, the practical implementation in Thailand, particularly in rural settings or among patients with limited home support may not offer the same degree of time-saving. In many cases, family members remain actively involved in device setup, monitoring, and troubleshooting. As a result, both informal caregiver costs (for daily life support) and formal caregiver costs were assumed to be similar between the APD and CAPD groups. We have added further explanation to the Discussion section to clarify this assumption.

*(Discussion, lines 341-345, page 17)*

*“In resource-limited settings such as Thailand, the practical advantages of APD may be diminished if patients rely heavily on family members for machine setup, monitoring, and troubleshooting. As a result, both informal caregiver costs for daily life support and formal caregiver costs were assumed to be similar between the APD and CAPD groups in our study.”*

7. The title and content of Table S1 should be revised: under the CAPD section, there are cost items related to APD and EPO per dose, which are not relevant in that context.

Response: Thank you for pointing out the inconsistency in Table S1. We agree that the inclusion of cost items related to APD and EPO per dose under the CAPD section was inappropriate and may have caused confusion. We have revised both the title and content of Table S1 to accurately reflect the relevant cost components for each modality and to improve overall clarity.

8. In line 213, under the one-way sensitivity analysis section, please specify which treatment the variable "number of weeks per year receiving treatment" refers to.

Response: Thank you for your comment. The variable “number of weeks per year receiving treatment” in the one-way sensitivity analysis refers to the assumed duration of dialysis treatment across all modalities. In the base-case analysis, we assumed 52 weeks of treatment per year for all groups, reflecting continuous year-round dialysis. A standard error of ±10% was applied to account variability. We have revised the manuscript to clarify this point, as shown in lines 256-257.

*“the number of weeks per year receiving dialysis treatment across all modalities”*

9. The results indicate that APD is the least favorable treatment among the three options, with lower quality of life and higher costs. This contradicts the expectations set in the introduction. While the authors cite previous findings from Thailand, are there any studies from other countries with different results? What could explain the reduced benefit of APD in the Thai population? This outcome also seems contrary to the current NHSO policy that prioritizes APD as the primary option.

Response: Thank you very much for raising this important point. We agree that the observed outcomes for APD may appear to contradict expectations set in the introduction and current policy directions. To address this, we have added further explanation in the Discussion section, including references to international studies with differing results and a discussion of contextual factors that may explain the reduced benefit of APD in the Thai setting.

*(Discussion, lines 329-353, pages 16-17),*

*“Moreover, patients in the APD group experienced a significantly greater decline in EQ-5D-5L scores over time compared to those in CAPD and CAPD+ICO groups, aligning with clinical findings that suggest poorer outcomes among APD patients in this context. While APD has been associated with greater convenience and improved HRQoL in high-income settings, evidence from other contexts has shown more variable results. For example, a prospective cohort study from the Netherlands, using data from the Netherlands Cooperative Study on the Adequacy of Dialysis (NECOSAD), found no statistically significant differences in HRQoL between patients receiving APD and those on CAPD over a three-year follow-up period [16]. Similarly, a study from* *Singapore reported only marginal improvements in specific physical health domains among APD patients, with no significant differences in overall HRQoL compared to CAPD [17]. These findings suggest that the benefits of APD may not be universally experienced. Variability in patient characteristics, availability of caregiver support, health system infrastructure, and dialysis practices may influence patient-reported outcomes. In resource-limited settings such as Thailand, the practical advantages of APD may be diminished if patients rely heavily on family members for machine setup, monitoring, and troubleshooting. As a result, both informal caregiver costs for daily life support and formal caregiver costs were assumed to be similar between the APD and CAPD groups in our study. This dependence can also reduce the perceived convenience and potentially impact quality of life. Although the current NHSO policy promotes APD as the preferred modality, our results demonstrate its relatively reduced cost-effectiveness. These findings are consistent with previous research conducted at Siriraj Hospital, which also found that APD was associated with higher lifetime costs and lower QALYs when compared to CAPD using glucose solution [18, 19]. However, the observed differences in cost and utility outcomes reflect the real-world implementation of APD in the Thai setting, where some expected advantages may not be fully realized or adequately captured. Therefore, future studies should explore on other potential benefits of APD within the Thai context.”*

10. In lines 296–297, please specify the year of the GDP reference used for comparison with the ICER values.

Response: We thank the reviewer for this important observation. The Gross Domestic Product (GDP) per capita used as a reference for comparing the ICER values in 2005 was based on Thailand’s GDP in 2005. We have revised the sentence as follows.

*(Discussion, lines 409-413, page 20).*

*“For instance, before the implementation of PD First policy, a study conducted in Thailand [22] found that compared to conservative treatment, both PD and HD were not cost-effective, with ICER values of 672,000 (1,933 USD) and 806,000 baht (23,189 USD) per QALY gained, equivalent to 2.5 and 3 times the gross domestic product (GDP) per capita in 2005, respectively [23].”*

11. It would be helpful for international readers if cost figures were presented in USD alongside Thai Baht.

Response: Thank you very much for your valuable suggestion. We have revised the manuscript to present all relevant cost figures in both Thai Baht and United States Dollars (USD) to enhance clarity and facilitate interpretation for international readers. This has been explained in the Methods section as follows.

*(Costs, lines 197-200, page 10).*

*“All costs were adjusted to 2023 values to ensure consistency within the same fiscal year, using the Consumer Price Index (CPI) for healthcare services and medications. Costs were then converted from Thai Baht to United States Dollars (USD) based on the 2023 exchange rate (1 THB = 0.02877 USD) [13].”*

12.To improve transparency and ensure adherence to international reporting standards, the authors are encouraged to follow the CHEERS 2022 checklist (Consolidated Health Economic Evaluation Reporting Standards). A completed checklist could be included as supplementary material.

Response: Thank you for your valuable suggestion. We have prepared and completed checklist and included it in the revised submission as supplementary material.

13. While revising your submission, please upload your figure files to the Preflight Analysis and Conversion Engine (PACE) digital diagnostic tool, https://pacev2.apexcovantage.com/. PACE helps ensure that figures meet PLOS requirements. To use PACE, you must first register as a user. Registration is free. Then, login and navigate to the UPLOAD tab, where you will find detailed instructions on how to use the tool. If you encounter any issues or have any questions when using PACE, please email PLOS at figures@plos.org. Please note that Supporting Information files do not need this step

Response: We have already uploaded our figure files to the Preflight Analysis and Conversion Engine (PACE) digital diagnostic tool.
