# Supplementary material for: Economic evaluation of dialysis treatment in end-stage renal disease patients with fluid and sodium overload: Evidence from a randomized controlled trial in Thailand
Source: PLoS One. 2025 Nov 5;20(11):e0335749. doi: 10.1371/journal.pone.0335749 (PMC12588515; doi:10.1371/journal.pone.0335749)
Supplement: Attachment — Submitted filename: Responses to Editors and Reviewers-17July25.docx [file pone.0335749.s007.docx]

**Responses to Editors and Reviewers**

1. Thank you for making revision for the previous comments. However, there are still some suggestions from our reviewer. Please address the Reviewer 1's comments:

"I welcome the revised version of the manuscript, which has significantly improved its readability and overall integrity. However, the authors' response to the reviewer’s concern regarding model validation and reliance on RCT data remains inadequate. They state that most key inputs are derived from the RCT but do not clarify whether the RCT was specifically designed or sufficiently powered to accurately estimate long-term parameters such as mortality or modality switching. Given that a single RCT often has limited sample sizes for capturing all relevant clinical indicators in economic evaluations, I recommend that the authors reference additional systematic reviews or other RCTs to compare results and substantiate their findings. Notably, studies from the Netherlands and Singapore cited in the manuscript show no significant difference in outcomes between CAPD and APD, contrasting with the clinical study used here, which suggests inferior outcomes for APD. Consequently, I suggest that the authors systematically identify and incorporate other RCTs or observational studies comparing life expectancy and quality of life between CAPD and APD to demonstrate that their clinical results are consistent and not outliers.”

Response: We thank the Reviewer 1 for the valuable comment and fully agree that relying solely on a single RCT for model inputs, particularly long-term parameters such as mortality and modality switching may be limiting. We much appreciate the opportunity to clarify and strengthen our approach. The primary RCT used in our model provided key short-term clinical data relevant to the local context; however, we acknowledge that it was not specifically powered to estimate long-term outcomes such as mortality and switching rates between dialysis modalities. To address this limitation and validate the robustness of our findings, we incorporated additional evidence from an external source. Specifically, we included data from a study conducted in Singapore by Yang et al. (2016), which provided mortality rates and EQ-5D–based utility values for patients receiving CAPD and APD. These parameters were applied in a scenario analysis to examine whether the cost-effectiveness conclusions held when using an external data. In addition, we also reviewed the study from the Netherlands (van de Luijtgaarden et al., 2011) as mentioned by the reviewer. However, this study did not report quality of life data based on EQ-5D or other preference-based measures necessary for calculating QALYs, and thus was not included in our scenario analysis. We thank the reviewer again for this constructive recommendation, which has helped improve the transparency, credibility, and generalizability of our model and its conclusions. Please see the Methods, Results, and Discussion sections of the manuscript which have been revised to reflect these additions as follows.

(Methods, Uncertainty analysis, page 11, lines 223-232)

*“As the RCT data only covered the first three years, we assumed a constant mortality rate beyond year 3 based on the average annual mortality observed in years 1–3. A scenario analysis was conducted to assess the impact of increasing the probability of death from year 3 onward (ranging from 0% to 100%) on the ICER values of CAPD+ICO and APD compared to glucose-based CAPD. Additionally, a scenario analysis was conducted using age-specific mortality rates and EQ-5D–based health utility values for patients receiving CAPD and APD, derived from an external study conducted in Singapore [16], to assess the robustness and external validity of the model. These inputs were applied to the same Markov model structure used in the base case. The purpose of this scenario analysis was to examine whether the model’s conclusions would remain consistent when applying alternative input data from a comparable regional setting.”*

(Results, Scenario analysis, page 15, lines 308-317)

*“In addition, scenario analysis was also conducted using age-specific mortality rates and EQ-5D–based health utility values from Yang et al.'s study in Singapore to assess the robustness of the model’s conclusions. Compared to the base case, the ICER for CAPD+ICO versus glucose-based CAPD increased substantially from 908,440 to 6,734,400 baht per QALY gained, reflecting a significant reduction in incremental QALYs (from 1.04 to 0.14), while incremental costs remained the same (Table S3). This suggests that CAPD+ICO is considerably less cost-effective under these external assumptions. APD remained dominated in both the base case and the scenario analysis, with higher costs and lower QALYs compared to glucose-based CAPD. Notably, the loss in QALYs with APD was more noticeable under the Singapore-based inputs (−0.48 vs. −0.22), further diminishing its cost-effectiveness.”*

(Discussion, page 19, lines 383-396)

*“However, our RCT study was not specifically powered to estimate long-term outcomes such as mortality and switching rates between dialysis modalities. Therefore, we conducted a scenario analysis incorporating mortality rates and EQ-5D–based utility values from a study conducted in Singapore by Yang et al.’s study [16]. The scenario analysis highlights that while the overall cost-effectiveness conclusions remained unchanged, the results were moderately sensitive to variations in mortality and health utility inputs. CAPD+ICO remained not cost-effective at the Thai societal WTP threshold, and APD continued to be dominated in both scenarios with even greater QALY losses under the external assumptions. However, the incremental LYs for APD increased from −0.14 in the base case to 0.18 in the scenario using Singaporean clinical and utility data. This change reflects improved survival assumptions derived from the Singaporean dataset, where patients on APD experienced better outcomes compared to those in the Thai RCT-based base case. These findings underscore the influence of data sources on model outcomes and highlight that contextual clinical effectiveness plays a role in determining LY gained and ultimately cost-effectiveness results.”*

2. Furthermore, the authors do not mention any formal validation or calibration of their model against external data or observational studies, such as comparing predicted mortality or modality switching rates with real-world evidence. Relying solely on face validation is insufficient when results are questionable—for example, the reported average life expectancy of 3-5 years for dialysis patients appears inconsistent with broader evidence. Incorporating formal validation exercises would strengthen the robustness and policy relevance of their findings.

Response: Thank you for your valuable comment. We agree that formal model validation is essential to enhance the credibility and policy relevance of economic evaluations. In response, we have now conducted an external validation by comparing the age-specific mortality rates projected by our model across the three dialysis modalities (CAPD, CAPD+ICO, and APD) with real-world data from a recently published Thai cohort study by Thuanman et al. (2024). This study followed 1,858 ESRD patients on PD who were hospitalized due to fluid overload between 2008 and 2018. This external validation result enhances confidence in the reliability of our model outcomes and provides reassurance that the predicted life expectancy of 3–5 years is in line with local empirical evidence. The comparison between modelled and observed mortality rates is now incorporated into the revised manuscript, with additional details provided in the Methods, Results, and Discussion sections. We have also included a new figure (“Fig. 6 Model validation results.”) to visually present the validation results.

(Methods, Model validation, page 11, lines 223-232)

*“To assess the validity of the model outputs, both face validation and external validation techniques were employed. Face validation was performed through consultation with clinical experts in nephrology and health economists to verify the model structure, assumptions, key inputs, and preliminary results ensuring clinical plausibility and alignment with real-world treatment pathways in Thailand. For external validation, model-predicted age-specific mortality rates across dialysis modalities (CAPD, APD, and CAPD+ICO) were compared with observed mortality data from a recently published cohort study conducted in Thailand by Thuanman et al. (2024), which investigated the survival of 1,858 ESRD patients on PD who were hospitalized due to fluid overload between January 2008 and December 2018 [17]. This comparison aimed to assess whether the modeled mortality trends were consistent with empirical real-world evidence.”*

(Results, Model validation, page 16-17, lines 337-344)

*“Fig. 6 illustrates the cumulative age-specific mortality rates ranging from 55–65 years for CAPD, CAPD+ICO, and APD. Overall, the modelled mortality curves show a consistent trend of increasing mortality with age, in line with the empirical data of a recent cohort study. During the earlier age intervals (55–58 years), the modelled mortality curves for APD and CAPD closely matched the real-world data, suggesting strong external validity of the modelled survival estimates of these modalities. In contrast, the CAPD+ICO strategy consistently projected lower cumulative mortality than observed in the empirical dataset, reflecting the model’s assumption of enhanced survival benefits with icodextrin use.”*

(Discussion, page 23-24, lines 495-506)

*“To enhance the external validity of our model, we conducted a formal model validation by comparing the modelled age-specific mortality rates for CAPD, CAPD+ICO, and APD with empirical data from a recent cohort study of Thai peritoneal dialysis patients who had been hospitalized due to fluid overload. The modelled survival estimates for CAPD and APD aligned well with the observed mortality rates, particularly in the earlier age intervals, supporting the robustness of our survival assumptions for these modalities. It is important to note that the patients in the cohort study who were hospitalized for fluid overload had higher mortality rates than those included in our RCT study, which may partly explain the differences observed. This highlights the need for additional real-world evidence to further refine survival assumptions in future model iterations. Overall, the validation results provide reassurance that the model is able to replicate key survival patterns seen in routine clinical practice, thus strengthening the credibility of our findings for informing dialysis policy decisions in Thailand.”*

3. While I appreciate the revised discussion, the authors’ mention of considerations such as health equity, clinical effectiveness, and patient-centered outcomes raises questions about whether these factors might influence policy recommendations differently from decisions based solely on economic evaluation. I encourage the authors to elaborate on these points to clarify how such broader considerations could impact decision-making processes. Providing additional context or examples, especially relevant to the Thai healthcare system and dialysis interventions, would help readers understand how these factors interplay with economic evidence in shaping policy decisions"

Response:

We appreciate the reviewer’s thoughtful observation and agree that health policy decisions often extend beyond economic evaluation alone. In response, we have expanded the Discussion section to elaborate on how broader considerations such as equity, patient-centered outcomes, and clinical feasibility interact with economic evaluation evidence in shaping policy decision in the Thai context, particularly for dialysis interventions.

(Discussion, page 21-22, lines 437-469)

*“From a purely cost-effectiveness perspective, our findings suggest that CAPD+ICO is not considered cost-effective compared to CAPD using glucose solution at a WTP threshold of 160,000 baht (4,603 USD) per QALY gained. Adopting CAPD+ICO as the standard modality would require an additional annual budget of approximately 58 million baht (2 million USD). However, it is important to note that patients receiving CAPD+ICO experienced improved clinical outcomes, including longer life expectancy and better HRQoL [12]. While these outcomes extend beyond the scope of a conventional cost-utility analysis, they are highly relevant in real-world policymaking, where decision-makers must balance economic efficiency with broader health system goals.*

*In Thailand, benefit package decisions are not based solely on cost-effectiveness [25]. Instead, the national health technology assessment (HTA) framework explicitly incorporates multiple criteria, including ethical, equity, and social considerations, as well as budget impact and system feasibility [26]. A key historical example is the adoption of the “PD First” policy in 2008, which mandated CAPD as the first-line dialysis modality under the UHC [27]. This decision was made despite cost-effectiveness analyses at the time indicating that both PD and HD were not cost-effective compared to conservative management, with ICERs of 672,000 baht (1,933 USD) and 806,000 baht (23,189 USD) per QALY gained, respectively [28]. These values were equivalent to 2.5 and 3 times the gross domestic product (GDP) per capita, exceeding the commonly used WTP threshold of one GDP per capita [29]. The policy was instead driven by concerns about equitable access to dialysis services, particularly for rural populations. CAPD was favored because it can be self-managed at home, unlike HD, which requires access to facility-based care concentrated in urban areas [30].*

*Our study's findings must also be interpreted in light of the recent policy shift toward an APD-first approach implemented by the NHSO in 2024. While this may affect the direct applicability of our results, the analysis remains relevant for informing future policy refinements, particularly for high-risk subgroups such as ESRD patients with fluid and sodium overload, for whom icodextrin-based therapies may provide additional clinical benefits. Our findings highlight the importance of incorporating broader decision-making criteria beyond cost-effectiveness alone. Clinical effectiveness, patient-reported outcomes, and health equity considerations particularly in a publicly funded system like Thailand can justify policy decisions that may not appear cost-effective when considered individually. Recognizing this complexity is essential for developing policies that are not only economically sound but also socially responsive and contextually appropriate.”*

4. Also, there was a minor suggestion from Reviewer 2: "I have only one remaining point: it may be helpful to include a supplementary table summarizing the baseline characteristics of the study participants (e.g., age, sex, comorbidities, degree of fluid overload, and relevant prescriptions). Providing this information would enhance the applicability and interpretability of the study findings."

Response: We appreciate Reviewer 2’s suggestion. We have addressed this point by including Table S4 in the supplementary materials, which summarizes the baseline characteristics of the study participants, including age, sex, comorbidities, degree of fluid overload, and relevant prescriptions. We believe this addition enhances the transparency and interpretability of our findings.
