# Supplementary material for: Economic evaluation of dialysis treatment in end-stage renal disease patients with fluid and sodium overload: Evidence from a randomized controlled trial in Thailand
Source: PLoS One. 2025 Nov 5;20(11):e0335749. doi: 10.1371/journal.pone.0335749 (PMC12588515; doi:10.1371/journal.pone.0335749)
Supplement: Attachment — Submitted filename: Responses to Editors and Reviewers-11Sep25.docx [file pone.0335749.s008.docx]

**Responses to Editors and Reviewers**

**Journal Requirements:**

1. If the reviewer comments include a recommendation to cite specific previously published works, please review and evaluate these publications to determine whether they are relevant and should be cited. There is no requirement to cite these works unless the editor has indicated otherwise.

Response: Thank you very much for your suggestion. We have carefully reviewed the publications recommended by the reviewer. Relevant studies that support or contextualize our findings have been cited in the revised manuscript. We did not include those that were not directly pertinent to our study objectives, in line with the journal’s guidance.

2. Please review your reference list to ensure that it is complete and correct. If you have cited papers that have been retracted, please include the rationale for doing so in the manuscript text, or remove these references and replace them with relevant current references. Any changes to the reference list should be mentioned in the rebuttal letter that accompanies your revised manuscript. If you need to cite a retracted article, indicate the article’s retracted status in the References list and also include a citation and full reference for the retraction notice. Please use the space provided to explain your answers to the questions above. You may also include additional comments for the author, including concerns about dual publication, research ethics, or publication ethics. (Please upload your review as an attachment if it exceeds 20,000 characters)

Response: Thank you very much for your suggestion. We have already reviewed our reference list and ensured that it is complete and correct.

**Reviewer #1:**

Thank you for your thorough revision and for thoughtfully addressing the points raised. I appreciate the inclusion of external data for scenario analysis and the validation against Thai cohort data—both of which enhance the credibility of your model. The expanded discussion on broader policy considerations, such as health equity and clinical effectiveness, adds valuable context, particularly within the Thai healthcare setting.

Response: Thank you very much for your insightful suggestions and kind feedback. We greatly appreciate your comments, and we believe that incorporating them has substantially improved the quality and clarity of our manuscript.

This version of the manuscript represents a substantial improvement and is close to being publication-ready. To further strengthen the work, I offer the following suggestions:

• Since the study is primarily based on a single clinical trial, I recommend revising the title to clearly reflect this, ensuring that the scope of the analysis is accurately represented.

Response: We sincerely appreciate the reviewer’s valuable suggestion regarding the title. We agree that the original title could imply a broader scope than the study actually covers, which is primarily based on a single clinical trial. To accurately reflect the study design and provide clarity for readers, we have revised the title as follows:

*“Economic evaluation of dialysis treatment in end-stage renal disease patients with fluid and sodium overload: evidence from a randomized controlled trial in Thailand”*

• Although mortality and utility parameters from the Singapore study are included in the sensitivity analysis, presenting these findings probabilistically—such as through cost-effectiveness acceptability curves—would be more informative than relying solely on deterministic ICERs.

Response: Thank you very much for your valuable suggestion. In response, we have presented the probabilistic sensitivity analysis (PSA) findings using cost-effectiveness acceptability curves (CEACs) for both the base-case (Figure 4) and the scenario using mortality and utility parameters from Yang et al.’s study in Singapore (Figure 5), thereby providing a more informative representation of decision uncertainty. Additional explanations have been included in the Results section (page 15-16, lines 316-320)

*“At Thailand’s WTP threshold of 160,000 THB (4,603 USD) per QALY gained, glucose-based CAPD had the highest probability of being cost-effective (87%), followed by APD (13%). When applying mortality and utility parameters from Yang et al.’s study, CAPD remained dominant with a 97% probability of cost-effectiveness, reinforcing the robustness of our conclusions across parameter variations.”*

• While I appreciate the additional discussion on the current dialysis policy in Thailand, please clarify the following sentence in a more explicit manner: "Our study's findings must also be interpreted in light of the recent policy shift toward an APD-first approach implemented by the NHSO in 2024. While this may affect the direct applicability of our results,...." Specifically, it would be helpful to elaborate on how this policy change might influence the relevance of your findings, as well as how your study’s results can inform or relate to the current policy, taking into account differences in patient populations, treatment pathways, or other relevant factors.

Response: Thank you for your valuable comment. We have revised the manuscript to clarify how the APD-first policy introduced by the NHSO in 2024 may influence the interpretation of our study findings and how our results can inform current policy discussions (Discussion, page 22-23, lines 464-477).

*“Our study's findings must also be interpreted in light of the recent policy shift toward an APD-first approach implemented by the NHSO in 2024. Although CAPD was identified as the most cost-effective option under Thailand’s WTP threshold, and APD was found to be dominated in the subgroup of patients with fluid and sodium overload, the APD-first policy reflects broader considerations beyond cost-effectiveness, including patient preference, service logistics, and workforce distribution. Importantly, the Thai HTA Guideline [14] emphasizes that policy decisions should not be based solely on cost-effectiveness but should also incorporate ethical implications, equity concerns, and social values. In this context, APD may not be cost-effective in certain subgroups but could still be justified for patients unable to perform CAPD independently or for those requiring greater flexibility to accommodate work or caregiving responsibilities. Thus, our findings remain relevant for informing refinement of the APD-first policy by identifying clinical contexts and patient subgroups where APD use is both clinically appropriate and economically reasonable, thereby supporting Thailand’s goals of universal health coverage, patient-centered care, and equitable resource allocation.”*

**Reviewer #2:**

Thank you for addressing my suggestion. Table S4 provides a clear and useful summary of the baseline characteristics and enhances the interpretability of the study. However, I recommend that the authors explicitly cite this supplementary table in the main text, ideally in the Methods section when describing the study population.

With this minor revision, I have no further concerns regarding the manuscript.

Response: Thank you for your valuable comment. We have cited Table S4 under Methods section (page 5, lines 101-102).
